# Anti-tumour promoter activity in Malaysian ginger rhizobia used in traditional medicine

**DOI:** 10.1038/sj.bjc.6690329

**Published:** 1999-04

**Authors:** S Vimala, A W Norhanom, M Yadav

**Affiliations:** Medicinal Plant Division, Forest Research Institute Malaysia, Kepong 52109, Kuala Lumpur; Centre for Foundation Studies in Science andDepartment of Genetics and Cellular Biology, University of Malaya, 50603 Kuala Lumpur, Malaysia

**Keywords:** anti-tumour promoters, Zingiberaceae, EBV-EA inhibition

## Abstract

Zingiberaceae rhizomes commonly used in the Malaysian traditional medicine were screened for anti-tumour promoter activity using the short-term assay of inhibition of 12-O-tetradecanoyl phorbol-13-acetate (TPA)-induced Epstein-Barr virus early antigen (EBV-EA) in Raji cells. The inhibition of TPA-induced EBV-EA was detected using the indirect immunofluorescence assay (IFA) and Western blot technique. The indirect IFA detected the expression/inhibition of EBV-EA-D (diffused EA antigen), whereas the Western blot technique detected the expression/inhibition of both EBV-EA-D and EA-R (restricted EA antigen). Seven rhizomes were found to possess inhibitory activity towards EBV activation, induced by TPA; they are: *Curcuma domestica*, *C*. *xanthorrhiza*, *Kaempferia galanga*, *Zingiber cassumunar*, *Z*. *officinale*, *Z*. *officinale* (red variety), and *Z*. *zerumbet*. A cytotoxicity assay was carried out to determine the toxicity of the Zingiberaceae rhizome extracts. The rhizome extracts that exhibited EBV activation inhibitory activity had no cytotoxicity effect in Raji cells. Therefore, the present study shows that several Zingiberaceae species used in Malaysian traditional medicine contain naturally occurring non-toxic compounds that inhibit the EBV activation, which, if further investigated, could contribute in the development of cancer prevention methods at the tumour-promoting stage. © 1999 Cancer Research Campaign


*British Journal of Cancer* (1999) **80**(1/2), 110–116
© 1999 Cancer Research Campaign
Article no. bjoc.1998.0329

### Anti-tumour promoter activity in Malaysian ginger rhizobia used in traditional medicine

**S Vimala[1], AW Norhanom[2] and M Yadav[3]**

[1]Medicinal Plant Division, Forest Research Institute Malaysia, Kepong 52109, Kuala Lumpur; [2]Centre for Foundation Studies in Science; [3]Department of Genetics and Cellular Biology, University of Malaya, 50603 Kuala Lumpur, Malaysia

**Summary** Zingiberaceae rhizomes commonly used in the Malaysian traditional medicine were screened for anti-tumour promoter activity using the short-term assay of inhibition of 12-O-tetradecanoyl phorbol-13-acetate (TPA)-induced Epstein-Barr virus early antigen (EBV-EA) in Raji cells. The inhibition of TPA-induced EBV-EA was detected using the indirect immunofluorescence assay (IFA) and Western blot technique. The indirect IFA detected the expression/inhibition of EBV-EA-D (diffused EA antigen), whereas the Western blot technique detected the expression/inhibition of both EBV-EA-D and EA-R (restricted EA antigen). Seven rhizomes were found to possess inhibitory activity towards EBV activation, induced by TPA; they are: *Curcuma domestica*, *C. xanthorrhiza*, *Kaempferia galanga*, *Zingiber cassumunar*, *Z. officinale*, *Z. officinale* (red variety), and *Z. zerumbet*. A cytotoxicity assay was carried out to determine the toxicity of the Zingiberaceae rhizome extracts. The rhizome extracts that exhibited EBV activation inhibitory activity had no cytotoxicity effect in Raji cells. Therefore, the present study shows that several Zingiberaceae species used in Malaysian traditional medicine contain naturally occurring non-toxic compounds that inhibit the EBV activation, which, if further investigated, could contribute in the development of cancer prevention methods at the tumour-promoting stage.

**Keywords:** anti-tumour promoters; Zingiberaceae; EBV-EA inhibition

Today, one of the most urgent problems of public health is the development of effective methods to block the carcinogenesis sequential events. Chemical carcinogenesis has been recognized to generally have three stages: initiation, promotion and progression (Hennings et al, 1983; Boutwell, 1989; Pitot and Dragan, 1991). Because a cure for cancer has not been established, cancer chemoprevention from edible plants may become a means to reduce cancer incidence.

Chemical chemoprevention is a concept defined as the prevention of cancer by the administration of natural or synthetic pure chemicals, or by daily foods enriched with cancer preventive components (Wattenberg, 1985; Greenwald et al, 1990; Weinstein, 1991; Morse and Stoner, 1993). Primary cancer prevention has two aspects in its methodology: (1) exclusion or avoidance of the environment carcinogens or other chemical factors closely relating to carcinogenesis such as tumour promoters; and (2) the administration of inhibitory or suppressive agents against carcinogenesis (Murakami et al, 1994). Because the continuous exposure to environmental and food carcinogens is inevitable in daily life, direct inhibition or suppression at the tumour-promoting stage could be an achievable strategy. Particularly, phytochemicals from edible plants would have an advantage in their clinical application on account of low toxicity. In fact, a great number of epidemiological studies of the relationship between food and cancer, together with research in experimental animal models, have demonstrated that daily ingestion of some vegetables and fruits could undoubtedly contribute to cancer prevention (Doll, 1992; Watternberg, 1992; Ziegler et al, 1992; Rogers et al, 1993).

Several compounds have been discovered with inhibitory effects on the tumour-promoting stage, and interestingly many of them were derived from plants. Koshimizu et al (1988) conducted screening tests of the methanol extracts from 121 species of Japanese vegetables and fruits for inhibitory activity toward Epstein–Barr virus early antigen (EBV-EA) activation induced by a 12-O-tetradecanoylphorbol-13-acetate (TPA) analogue 12-O-hexadecanoylphorbol-13-acetate (HPA). As a result, 26% of the total showed significant inhibitory activity (>30% at 200 $\mu g\ ml^{-1}$). These results show that anti-tumour promoters occur in a wide variety of plants.

The Zingiberaceae is a well-known plant family in Southeast Asia and numerous of its species are being used in traditional medicine, which is found to be quite effective in the treatment of several diseases. In Malaysia, various ginger rhizobia are widely used in the traditional treatment of ailments such as stomach problems, nausea, vomiting, epilepsy, sore throat, cough, bruises, wounds, childbirth, eyewash, sore eyes, liver complaints, rheumatism, muscular pains, ringworm, asthma, fever, malignancies, swelling and several other disorders (Burkill, 1966).

*Curcuma domestica*, *Kaempferia galanga* and *Languas galanga* from the Zingiberaceae family have been reported to demonstrate anti-tumour promoter activity (Koshimizu et al, 1988; Nishino et al, 1990). Several other ginger species are used in Malaysian traditional medicine, but their anti-tumour promoter activity has not been previously investigated. Thus, further examination of the Zingiberaceae species on their anti-tumour-promoting activity was worthwhile undertaking. The primary objective of this study was

*Received 15 January 1997*
*Revised 30 June 1998*
*Accepted 2 July 1998*

*Correspondence to:* S Vimala to screen several species of the Zingiberaceae family most frequently used in local traditional medication for the presence of anti-tumour promoter activity. The short-term assay of inhibition of TPA-induced EBV-EA in Raji cells was used to identify potential anti-tumour promoter activity in seven Malaysian ginger rhizobia.

Human lymphoblastoid Raji, a non-producer cell line which harbours multiple copies of EBV genomes (Ernberg and Klein, 1979), can be activated by tumour promoters such as TPA to express EBV-EA (Kawanishi and Ito, 1982). Zur Hausen et al (1978, 1979) demonstrated that phorbol esters which possessed tumour-promoting activity could induce viral cycle in latently infected cells carrying EBV, and Ito et al (1981*a*) noted that low concentration of *n*-butyrate increased the effects of tumour promoters synergistically. It is noteworthy that such EBV-activating principles can be considered to possess tumour-promoting capacity (Ito et al, 1981*b*). However, extracts of certain plant species were found to inhibit the TPA-induced EBV-EA activation. Thus, this assay system of inhibition of EBV-EA activation by TPA in indicator Raji cells is regularly used as an early method of detecting anti-tumour-promoting principles in natural products.

Inhibitory potentials against tumour promoter-induced EBV activation has been reported to be well correlated with those in some animal models (Murakami et al, 1996). Ursolic acid and mokko lactone, isolated from green perrilla and edible burdok extracts respectively, were found to inhibit EBV-EA activation induced by HPA (Koshimizu et al, 1988), and proven to have anti-tumour-promoting activity in ICR mice skin (Koshimizu and Ohigashi, 1991). Several other active components from common food that inhibited the TPA-induced EBV-EA activation (Ohigashi et al, 1986) were also shown to inhibit skin tumour formation by TPA in 7,12-dimethylbenz[a]anthracene-initiated mice (Tokuda et al, 1986).

Some of the naturally occurring anti-tumour promoters identified in edible Thai plants by the inhibitory test of EBV-EA activation, induced by 40 ng $ml^{-1}$ HPA (Murakami et al, 1995*a*), have further been proven to possess anti-tumour-promoting activity in vivo (Murakami et al, 1995*b*). Ohigashi et al (1992) screened marine algae, which is claimed to reduce cancer risk in Japan, for EBV-EA activation inhibitory activity and the dichloromethane of *Undaria pinnatifida* that showed strong inhibitory activity (>70% at 4 µg $ml^{-1}$) was also found to completely suppress tumour formation on mouse skin induced by DMBA and TPA. The acetone extract of *Scutellaria baicalensis* that showed significant inhibitory effects on EBV-EA activation in vitro was fractionated with ethyl acetate and two flavones, 5,7,2′-trihydroxyflavone and 5,7,2′,3′-tetrahydroxyflavone were found to exhibit strong inhibitory effect on mouse skin tumour promotion in an in vivo two-stage carcinogenesis test (Konoshima et al, 1992).

The above findings show that the EBV genome carrying human lymphoblastoid cells (Raji), activated by tumour promoters, has been used widely as a screening assay for the early detection of anti-tumour promoters. In our search for natural inhibitors against tumour promotion, we screened eleven ginger rhizomes commonly found as ingredients in the Malaysian traditional medicine using the inhibition of TPA-induced EBV-EA activation assay.

### MATERIALS AND METHODS

### Plant material

Rhizomes of eleven species from the Zingiberaceae family, *Curcuma aeruginosa* (*temu hitam*), *Curcuma domestica* (turmeric/*kunyit*), *Curcuma mangga* (*temu pauh*), *Curcuma xanthorrhiza* (*temu lawak*), *Gastrochilus panduratum* (*temu kunci*), *Kaempferia galanga* (*chekur*), *Languas galanga* (*lengkuas*), *Zingiber cassumunar* (*bonglai*), *Zingiber officinale* (ginger/*halia*), *Zingiber officinale* (red variety) (*halia bara*) and *Zingiber zerumbet* (*lempoyang*), were collected from the University of Malaya botanical garden, Rimba Ilmu.

### Preparation of crude extracts

The Zingiberaceae rhizomes were extracted by the method of Ito et al (1983). Five grams of dried, ground rhizome was extracted using petroleum ether, chloroform and ethanol solvents successively. The powdered rhizome was soaked for 3 days in 20 ml of solvent. On day 3, the extract was removed from the plant debris and subjected to evaporation. The dry crude extract obtained was dissolved in DMSO (Sigma) to provide a stock solution of 20 mg $ml^{-1}$ and stored at –20°C. The plant debris were dried and resoaked with the following solvent.

### EBV early antigen (EA) induction in Raji cells

The technique used was as described previously (Ito et al, 1981*a*; Yadav et al, 1989). Raji cells were obtained from the National Cancer Institute, Bethesda, MD, USA (courtesy of Dr DV Ablashi). The culture medium consisted of RPMI-1640 medium supplemented with L-glutamine (0.2 g $l^{-1}$), inactivated fetal calf serum (10%), streptomycin (100 µg $ml^{-1}$) and penicillin (100 IU $ml^{-1}$). Rapidly growing human lymphoblastoid Raji cells in log phase at a density of $1 \times 10^6$ cells $ml^{-1}$ were incubated with plant extracts at concentrations ranging from 20 to 640 µg $ml^{-1}$ in the presence of 20 ng $ml^{-1}$ TPA (Sigma) and 4 mmol $ml^{-1}$ sodium *n*-butyrate (Sigma). The cells were incubated for 72 h in a humidified incubator at 37°C with 5% carbon dioxide in air, without change of media. Previous studies showed that optimum EA activity was obtained after 72 h of incubation (Yadav et al, 1989). The positive controls consisted of untreated culture media and Raji cells treated with 4 mmol $ml^{-1}$ sodium *n*-butyrate. The negative control consisted of Raji cells treated with optimal concentration, 20 ng $ml^{-1}$ of TPA and 4 mmol $ml^{-1}$ sodium *n*-butyrate. The assay was repeated once to obtain consistent results.

### Indirect immunofluorescence assay (IFA)

To observe the EBV-EA expression in the treated and fixed cells, a modification of the indirect IFA was used (Henle and Henle, 1966; Henle et al, 1971). EBV EA-positive serum, obtained from nasopharyngeal carcinoma (NPC) patients, with titre values of 1:640 and 1:1280 were used to detect the presence of EBV-EA. Twenty microlitres of the serum, at a dilution of 1:20, was dispensed into each well of the multitest slide containing acetone-fixed Raji cells. The slides were then incubated in a humidity chamber at 37°C for 45 min. At the end of the incubation period, the slides were washed with phosphate-buffered saline (PBS) and 20 µl of fluorescein thiocynate-conjugated (FITC) IgG, at a dilution of 1:20, was added to each well. The slides were reincubated and washed as above and air dried.

### Detection of EBV activation inhibitory activity

The fluorescence of the cells were observed using a UV microscope (Olympus, Japan). The control cells, both positive and negative, were routinely incorporated in each assay.

© Cancer Research Campaign 1999

Treatment with increasing concentrations of plant extracts decreased the number of fluorescent cells. Hence, the first well which showed no fluorescent cells was taken as the concentration and titre for complete EBV activation inhibitory activity [inhibitory effect (IE): 100%].

EBV activation was measured by detection of EA. The ratio of EA-induced cells was compared with that of a control experiment only with sodium *n*-butyrate and TPA, in which the ratio of EA-induced cells was ordinarily around 50%. The control was considered as 100% tumour promotion and 0% inhibition.

### Anti-tumour promoter standard

Ascorbic acid (1000 μM; Sigma), a known anti-tumour promoter, was used as a standard for method validation. It showed 50% inhibition against TPA-induced EBV-EA activation in Raji cells compared with negative control and 0% cytotoxicity relative to controls.

### Cytotoxicity assay

Rapidly growing human lymphoblastoid Raji cells were incubated as mentioned above. On day 3, the cells were spun down at 1000 r.p.m for 5 min and 0.1% of Trypan Blue was added to the cell suspension. Cell enumeration was carried out using a haemocytometer.

### Detection of the EA protein suppression (Western blot)

The EBV-EA protein suppression was detected by the Western blot technique (Towbin et al, 1979; Burnette, 1981). Gel electrophoresis was carried out according to the method of Laemmli (1970). Bio-Rad prestained standards of both low and high range were used as markers. The protein bands were transferred to a piece of Immobilon PVDF transfer membrane in a semidry transfer cell (Bio-Rad, USA) using a constant current of 0.5 mA for a period of 1 h. Then the transfer membrane was removed from the sandwich and incubated in 0.1 ml of blocking solution per $cm^2$ of the membrane for 1–2 h at room temperature with gentle agitation. The blocking solution consisted of 5 g of non-fat milk (Oxoid, UK) and 20 μl of Tween-20 (Merck, Germany) in 100 ml of PBS. The membrane was then incubated overnight with NPC serum diluted in the blocking solution (1:200) at 4°C with gentle agitation. The following morning, the membrane was washed three times and incubated in 0.5 μg $ml^{-1}$ of enzyme-coupled secondary reagent, affinity purified goat anti-human (H&L) IgG horseradish peroxidase conjugate (Bio-Rad, USA) for 1 h at room temperature with gentle agitation. The membrane was then immersed in the colour development solution (Bio-Rad) containing chromogenic substrate with enzyme-coupled antibody and incubated at room temperature with gentle agitation for about 2–3 min till the protein bands were clearly visible.

### RESULTS

Eleven ginger rhizomes were screened for anti-tumour promoter activity using the indirect IFA and Western blot technique, and the results obtained from these two techniques were compatible.

The indirect IFA detected the inhibition of TPA-induced EBV-EA-D (diffused EA complex) in Raji cells. The positive controls, which consisted of untreated Raji cells and Raji cells treated with 4 mmol $ml^{-1}$ sodium *n*-butyrate, demonstrated non-fluorescing cells, i.e. no expression of the EBV-EA. The negative control that consisted of Raji cells treated with the combination of 20 ng $ml^{-1}$ TPA and 4 mmol $ml^{-1}$ sodium *n*-butyrate showed 100% expression of the EBV-EA through fluorescing cells that were detected by the indirect IFA. As shown in Table 1, seven ginger rhizomes were found to express EBV activation inhibitory activity when tested at

**Table 1** Inhibition (%) of TPA-induced EBV-EA activation (IFA)

| Plant (vernacular name) | Extract | Extract concentration (μg $ml^{-1}$) | | | | | |
|---|---|---|---|---|---|---|---|
| | | 20 | 40 | 80 | 160 | 320 | 640 |
| *Curcuma domestica* | Petroleum ether | 100 | 100 | 100 | ct | ct | ct |
| *(kunyit)* | Chloroform | 100 | 100 | 100 | 100 | 100 | 100 |
| | Ethanol | 100 | 100 | 100 | 100 | 100 | 100 |
| *Curcuma xanthorrhiza* | Chloroform | 0 | 100 | ct | ct | ct | ct |
| *(temu lawak)* | Ethanol | 0 | 0 | 66 | 100 | ct | ct |
| *Kaempferia galanga* | Ethanol | 0 | 0 | 0 | 0 | 80 | 90 |
| *(chekur)* | | | | | | | |
| *Zingiber cassumunar* | Petroleum ether | 100 | 100 | 100 | ct | ct | ct |
| *(bonglai)* | Chloroform | 100 | 100 | 100 | 100 | 100 | 100 |
| | Ethanol | 0 | 80 | 100 | 100 | 100 | 100 |
| *Zingiber officinale* | Petroleum ether | 0 | 0 | 100 | 100 | ct | ct |
| *(halia)* | Chloroform | 0 | 100 | 100 | 100 | ct | ct |
| | Ethanol | 0 | 0 | 81.7 | 100 | ct | ct |
| *Zingiber officinale* | Petroleum ether | 0 | 0 | 100 | ct | ct | ct |
| (red variety) | Chloroform | 0 | 100 | 100 | 100 | ct | ct |
| *(halia bara)* | Ethanol | 0 | 0 | 0 | 88 | 96 | 100 |
| *Zingiber zerumbet* | Chloroform | 100 | 100 | 100 | 100 | ct | ct |
| *(lempoyang)* | Ethanol | 0 | 0 | 70 | 96.7 | 100 | ct |

100%, complete inhibition; 99–1%, partial inhibition; 0%, no inhibition; ct, cytotoxic. Data, expressed as percentage of EA inhibition, are mean values of duplicate experiments (s.d.<5%).

© Cancer Research Campaign 1999

**Table 2** EBV-EA protein suppression in Raji cells detected by Western blot

| Plant (vernacular name) | Extract | Extract concentration (μg ml⁻¹) 20 | 40 | 80 | 160 | 320 | 640 |
|---|---|---|---|---|---|---|---|
| *Curcuma domestica* | Petroleum ether | + | + | + | ct | ct | ct |
| *(kunyit)* | Chloroform | + | + | + | + | + | + |
| | Ethanol | + | + | + | + | + | + |
| *Curcuma xanthorrhiza* | Chloroform | – | + | ct | ct | ct | ct |
| *(temu lawak)* | Ethanol | – | – | ± | + | ct | ct |
| *Kaempferia galanga* *(chekur)* | Ethanol | – | – | – | – | ± | ± |
| *Zingiber cassumunar* | Petroleum ether | + | + | + | ct | ct | ct |
| *(bonglai)* | Chloroform | + | + | + | + | + | + |
| | Ethanol | – | ± | + | + | + | + |
| *Zingiber officinale* | Petroleum ether | – | – | + | + | ct | ct |
| *(halia)* | Chloroform | – | + | + | + | ct | ct |
| | Ethanol | – | – | ± | + | ct | ct |
| *Zingiber officinale* | Petroleum ether | – | – | + | ct | ct | ct |
| (red variety) | Chloroform | – | + | + | + | ct | ct |
| *(halia bara)* | Ethanol | – | – | – | ± | ± | + |
| *Zingiber zerumbet* | Chloroform | + | + | + | + | ct | ct |
| *(lempoyang)* | Ethanol | – | – | ± | ± | + | ct |

+, Complete protein suppression; ±, partial protein suppression; –, no protein suppression; ct, cytotoxic. Observational data were derived from duplicate experiments.

concentrations of 20–640 μg ml$^{-1}$ cell culture medium. They were *C. domestica*, *C. xanthorrhiza*, *Z. officinale*, *Z. officinale* (red variety), *Z. cassumunar*, *Z. zerumbet* and *K. galanga*.

Similar results were obtained with the Western blot technique that detected the suppression of TPA-induced EBV-EA-D and EA-R (restricted EA complex), as shown in Table 2. The positive controls did not express any EA protein bands whereas the negative control was found to express the EA-D (47–56 kDa) and EA-R (85 kDa) components. An example of EA protein suppression by the petroleum ether extract of *Z. officinale* at concentrations 80–640 μg ml$^{-1}$ is shown in Figure 1.

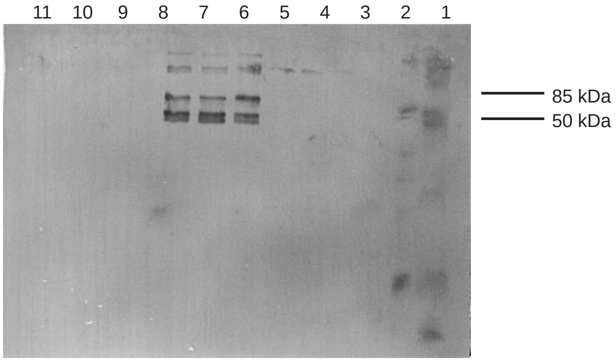

**Figure 1** Western Blot with petroleum ether extract of *Z. officinale* rhizome. A 12% SDS polyacrylamide gel electrophoresis (PAGE) run, transferred to Immobilon PVDF in the semidry Trans-Blot cell. Blot was treated with NPC (EA+) serum as primary antibody and goat anti-human IgG HRP conjugate as secondary antibody. The colour reaction was carried out using HRP conjugate substrate. Loading pattern: lane 1, prestained SDS-PAGE standard, low range; lane 2, prestained SDS-PAGE standard, high range; lane 3, untreated Raji cells, positive control; lane 4, Raji + 4 mmol ml$^{-1}$ sodium *n*-butyrate, positive control; lane 5, Raji + n-b + 20 ng ml$^{-1}$ TPA, negative control; lane 6, Raji + n-b + TPA + 20 μg ml$^{-1}$ plant extract; lane 7, Raji + n-b + TPA + 40 μg ml$^{-1}$ plant extract; lane 8, Raji + n-b + TPA + 80 μg ml$^{-1}$ plant extract; lane 9, Raji + n-b + TPA + 160 μg ml$^{-1}$ plant extract; lane 10, Raji + n-b + TPA + 320 μg ml$^{-1}$ plant extract; lane 11, Raji + n-b + TPA + 640 μg ml$^{-1}$ plant extract. The blot shows the EBV EA-D (47–56 kDa) and EA-R (85 kDa) protein bands, which were identified in lanes 5, 6 and 7 and were suppressed in lanes 8–11

A cytotoxicity assay was carried out to determine the toxicity of the ginger extracts in Raji cells. The controls that consisted of untreated Raji cells, Raji cells treated with 4 mmol ml$^{-1}$ sodium *n*-butyrate and Raji cells treated with the combination of 20 ng ml$^{-1}$ TPA and 4 mmol ml$^{-1}$ sodium *n*-butyrate showed no signs of cytotoxicity. These controls had dividing Raji cells at a density of $1 \times 10^6$ cells ml$^{-1}$. The percentage of killing shown in Table 3 is relative to these controls in which treatment with ginger extracts that caused cell death relatively more than controls were considered cytotoxic.

*C. domestica*, *Z. cassumunar* and *Z. zerumbet* showed strong inhibitory activity against TPA-induced EBV-EA activation. At all concentrations tested (20–640 μg ml$^{-1}$), the chloroform and ethanol extracts of *C. domestica* and the chloroform extract of *Z. cassumunar* demonstrated complete inhibitory activity (IE = 100%). The chloroform extract of *Z. zerumbet* showed complete inhibitory activity (IE = 100%) at concentrations 20–160 μg ml$^{-1}$. The petroleum ether extracts of *C. domestica* and *Z. cassumunar* showed complete inhibitory activity (IE = 100%) at concentrations 20–80 μg ml$^{-1}$. The ethanol extracts of *Z. cassumunar* and *Z. zerumbet* demonstrated 70–100% IE at concentrations 40–640 μg ml$^{-1}$. At these extract concentrations that inhibited EBV activation significantly, no cytotoxicity (CT) was observed in Raji cells (CT = 0%). However, the petroleum ether extract of *Z. zerumbet* was found to be toxic to Raji cells at all concentrations used.

*Z. officinale* and its red variety demonstrated EBV-EA inhibitory activity at higher concentrations. Complete inhibition (IE = 100%) was observed at 40–160 μg ml$^{-1}$ with treatment of

© Cancer Research Campaign 1999

**Table 3** Percentage killing of Raji cells (cytotoxicity assay)

| Plant (vernacular name) | Extract | Extract concentration (µg ml$^{-1}$) 20 | 40 | 80 | 160 | 320 | 640 |
|---|---|---|---|---|---|---|---|
| *Curcuma domestica* | Petroleum ether | 0 | 0 | 0 | 90 | 97 | 100 |
| *(kunyit)* | Chloroform | 0 | 0 | 0 | 0 | 0 | 0 |
| | Ethanol | 0 | 0 | 0 | 0 | 0 | 0 |
| *Curcuma xanthorrhiza* | Chloroform | 0 | 0 | 90 | 95 | 98 | 100 |
| *(temu lawak)* | Ethanol | 0 | 0 | 0 | 0 | 90 | 100 |
| *Kaempferia galanga (chekur)* | Ethanol | 0 | 0 | 0 | 0 | 0 | 0 |
| *Zingiber cassumunar* | Petroleum ether | 0 | 0 | 0 | 75 | 80 | 100 |
| *(bonglai)* | Chloroform | 0 | 0 | 0 | 0 | 0 | 0 |
| | Ethanol | 0 | 0 | 0 | 0 | 0 | 0 |
| *Zingiber officinale* | Petroleum ether | 0 | 0 | 0 | 0 | 100 | 100 |
| *(halia)* | Chloroform | 0 | 0 | 0 | 0 | 100 | 100 |
| | Ethanol | 0 | 0 | 0 | 0 | 100 | 100 |
| *Zingiber officinale* | Petroleum ether | 0 | 0 | 0 | 90 | 98 | 100 |
| (red variety) | Chloroform | 0 | 0 | 0 | 0 | 95 | 100 |
| *(halia bara)* | Ethanol | 0 | 0 | 0 | 0 | 0 | 100 |
| *Zingiber zerumbet* | Chloroform | 0 | 0 | 0 | 0 | 90 | 100 |
| *(lempoyang)* | Ethanol | 0 | 0 | 0 | 0 | 0 | 60 |

0, No cytotoxicity relative to controls. Data, expressed as percentage of killing, are mean values of duplicate experiments (s.d.<5%).

their chloroform extracts and 80–160 µg ml$^{-1}$ with petroleum ether extracts. At 20 µg ml$^{-1}$, no inhibitory activity was observed (IE = 0%), and at concentrations above 160 µg ml$^{-1}$ cytotoxicity was observed (CT = 90–100%). *C. xanthorrhiza* demonstrated 100% IE at 40 µg ml$^{-1}$ and 160 µg ml$^{-1}$ with treatment of the chloroform and ethanol extracts, respectively, however at higher concentrations cytotoxicity was observed (CT = 90–100%). At 20 µg ml$^{-1}$, no inhibitory effect was observed with these extracts (IE = 0%). The petroleum ether extract of *C. xanthorrhiza* did not inhibit EA activation at 20 µg ml$^{-1}$ and was toxic to Raji cells at all other concentrations.

The ethanol extract of *K. galanga* demonstrated partial inhibition (IE = 80–90%) at concentrations of 320–640 µg ml$^{-1}$. At lower concentrations of 20–80 µg ml$^{-1}$, no inhibition was observed (IE = 0%) with all three extracts. At concentrations of 160–640 µg ml$^{-1}$, cytotoxicity was observed with the treatment of petroleum ether and chloroform extracts.

Four other Zingiberaceae species, *C. aeruginosa*, *C. mangga*, *G. panduratum* and *L. galanga*, caused cell death (CT = 60–100%) because of toxicity at all concentrations tested, thus the EBV-EA inhibitory activity could not be determined.

### DISCUSSION

In the present study, 7 out of 11 ginger rhizomes tested were found to possess EBV-EA inhibitory activity with no cytotoxicity effect in Raji cells. They are *C. domestica*, *C. xanthorrhiza*, *Kaempferia galanga*, *Zingiber cassumunar*, *Z. officinale*, *Z. officinale* (red variety) and *Z. zerumbet*.

*C. domestica* or turmeric, which showed strong EA inhibitory activity, has had an extremely long use among the Malaysians (Burkill, 1966). It is the most frequently used species in the preparation of Malaysian traditional medicine, and is also used as spice, food preservative and cooking ingredient. In a previous study, curcumin, a principal in *C. domestica*, was found to suppress the action of tumour promoters in vitro and in vivo (Nishino et al, 1990). Curcumin inhibited TPA-enhanced phospholipid metabolism in cultured cells and was demonstrated to have anti-tumour-promoting activity in mouse skin carcinogenesis induced by TPA, however further experiments revealed that various kinds of anti-tumour promoter agents are mixed in the *C. domestica* rhizome (Nishino et al, 1990).

The chloroform extracts of *Z. cassumunar* and *Z. zerumbet* showed strong EA inhibitory activity, similar to *C. domestica*, showing that they possess active components as potent as *C. domestica*. *Z. cassumunar* and *Z. zerumbet* are cultivated in Malaysia for medicinal purposes and are found in the prescriptions of various traditional medicines (Burkill, 1966).

*Kaempferia galanga* rhizome that exhibited partial EA inhibitory activity is a favourite among the Malaysians who call it chekur. It is cultivated and is used medicinally to treat children and as a cooking ingredient (Burkill, 1966). In a previous study, the extract of *K. galanga* rhizome showed inhibition on TPA-stimulated phospholipid synthesis (Nishino et al, 1990).

*Z. officinale* is another widely used species of the Zingiberaceae family. It is a common additive in a large number of compounded foods and beverages because of its flavour and pungency. *Z. officinale* and its red variety are common ingredients in the Malaysian traditional medicine, but little has been studied on their anti-tumour-promoting activity. In this study, both the ginger varieties were found to demonstrate EA inhibitory activity. *C. xanthorrhiza*, popularly found in traditional tonic preparation (Burkill, 1966), also demonstrated EA inhibitory activity.

It is worthwhile to note that the seven ginger rhizobia found to possess EBV-EA inhibitory activity in the present study are common ingredients in food, tonics and traditional preparations that have been consumed over the years, indicating that they are probably free from toxicity and side-effects. The present results that showed no cytotoxicity effect in Raji cells at concentrations that completely inhibited EA activation support this assumption.

© Cancer Research Campaign 1999

Epidemiological studies have identified populations less prone to cancer, suggesting that environmental factors including diet may have an important contributory role (Doll, 1992; Watternberg, 1992; Ziegler et al, 1992; Rogers et al, 1993).

In cancer prevention, it would be effective to block the three stages of carcinogenesis independently or concurrently. However, it is worthwhile searching for effective anti-tumour promoters because the promotion stage is known to need a long-term period for completion and is the only process possessing reversibility (Berenbelum, 1975). Pitot and Sirica (1980) showed evidence that pure initiation caused irreversible change but not neoplasm unless a promoter is applied. Thus, the crucial step in the development of human cancer may be the second stage of carcinogenesis, i.e. the tumour promotion stage and not the first initiation stage. Epidemiological evidence argues strongly that tumour promotion is a significant factor in the final development of cancer in humans (Hammond, 1975).

At present, cancer patients treated with chemical chemopreventive compounds, which effectively suppress the evolution of the neoplastic process, are also exposed to the permissible risk of toxicity from the chemopreventive agent that increases with the dosage (Wattenberg, 1985). Findings in the present study, however, showed that the ginger rhizobia extracts which showed strong EBV-EA inhibitory activity did not cause any cytotoxicity effect in Raji cells.

Populations with high risk of cancer could be encouraged to take in their diets plants with anti-tumour-promoting principles. Although such an approach may not totally eliminate the disease, it could form one method of control. However, such methods have not been previously assessed and the efficacy of appropriate diets in control of cancer need to be investigated.

In the case of these ginger plants which have been studied, further investigations are being carried out to isolate and identify the active compounds in each and to demonstrate that they possess strong anti-tumour promoter activity in in vivo experiments. The detection and further analysis of these natural compounds could be useful in the development of a formulation for the dietary control of malignancies.

### ACKNOWLEDGEMENT

We would like to thank the University of Malaya for their financial assistance and support through Vote-F.

### REFERENCES

Berenbelum I (1975) Two-stage carcinogenesis. *Cancer* **1**: 323–344

Boutwell RK (1989) Model systems for defining initiation, promotion and progression of skin neoplasms. In *Skin Carcinogenesis*, Slaga TJ, Klein-Szanto AJP, Boutwell RK, Stevenson DE, Spitzer HL and D'Motto B (eds), pp. 3–15. Alan R Liss: New York

Burkill IH (1966) *A Dictionary of the Economic Products of the Malay Peninsula*, Vols 1 and 2. Ministry of Agriculture and Cooperative: Kuala Lumpur

Burnette WN (1981) Western blotting: electrophoretic transfer of proteins from sodium dodecylsulfate-polyacrlamide gels to unmodified nitrocellulose and radiographic detection with antibody and radioiodinated protein. *A Anal Biochem* **112**: 195–203

Doll SR (1992) The lessons of life: keynote address to the nutrition and cancer conference. *Cancer Res* **44**: 2024–2029

Ernberg I and Klein G (1979) EB virus-induced antigens. In *The Epstein–Barr virus*, Epstein MA and Achong BG (eds), pp. 40–52. Springer Verlag: Berlin

Greenwald P, Nixon DW, Malone WF, Kelloff GJ, Stern HR and Witkin KM. (1990). Concepts in cancer chemoprevention research. *Cancer* **65**: 1483–1490.

Hammond EC (1975) Tobacco. In *Persons at High Risk of Cancer*. Fraumeni Jr JF (ed.), pp. 131–138. Academic Press: New York

Henle G and Henle W (1966) Immunofluorescence in cells derived from Burkitt's lymphoma. *J Bacteriol* **91**: 1248–1236

Henle G, Henle W and Klein G (1971) Demonstration of two distinct components in the early antigen complex of Epstein–Barr virus infected cells. *Int J Cancer* **8**: 272–278

Hennings H, Shores R, Wenk ML, Spangler EF, Tarone R and Yuspa SH (1983) Malignant conversion of mouse skin tumours is increased by tumour initiators and not tumour promoters. *Nature* **304**: 67–69

Ito Y, Kawanishi M, Harayama T and Takabayashi S (1981*a*) Combined effect of the extracts from *Croton tiglium, Euphorbia lathyris* or *Euphobia tirucalli* and *n*-butyrate on Epstein–Barr virus expression in human lymphoblastoid P3HR-1 and Raji cells. *Cancer Lett* **12**: 175–180

Ito Y, Yanase S, Fujita J, Hirayama T, Takashima M and Imanaka H (1981*b*) A short-term in vitro assay for promoter substances using human lymphoblastoid cells latently infected with Epstein–Barr virus. *Cancer Lett* **13**: 29–37

Ito Y, Yanushe S, Tokuda H, Kishishita M, Chigashi H, Hirota M and Koshimizu K (1983) Epstein–Barr virus activation by Tung oil, extracts of *Aleurites fordii* and its diterpene ester 12-O-hexadecanoyl-16-hydroxy-phorbol-13-acetate. *Cancer Lett* **18**: 87–95

Kawanishi M and Ito Y (1982) Similarity of Epstein–Barr virus early polypeptides induced by various tumour promoters. *Cancer Lett* **16**: 19–23

Konoshima T, Kokumai M, Kozuka M, Iinuma M, Mizuno M, Tanaka T, Tokuda H, Nishino H and Iwashima A (1992) Studies on inhibitors of skin tumour promotion XI: inhibitory effects of flavonoids from *Scutellaria baicalensis* on Epstein–Barr virus activation and their anti-tumour promoting activities. *Chem Pharm Bull* **40**: 531–533

Koshimizu K, Ohigashi H, Tokuda H, Kondo A and Yamaguchi K (1988) Screening of edible plants against possible anti-tumour promoting activity. *Cancer Lett* **39**: 247–257

Koshimizu K and Ohigashi H (1991) Search for naturally occurring anti-tumour promoters by inhibition of tumour promoter-induced Epstein–Barr virus activation. In *Advances in New Drug Development*, Kim BK, Lee EB, Kim CK and Han YN (eds), pp. 438–447. Harl Rim Won Printing Co.: Seoul

Laemmli UK (1970) Cleavage of structural proteins during the assembly of the bacteriophage T4. *Nature* **227**: 680

Morse MA and Stoner GD (1993) Cancer prevention: principles and prospects. *Carcinogenesis* **14**: 1737–1746

Murakami A, Ohigashi H and Koshimizu K (1994) Possible anti-tumour promoting properties of traditional Thai food items and some of their active constituents. *As Pac J Clin Nutr* **3**: 185–191

Murakami A, Jiwajinda S, Koshimizu K (1995*a*). Screening for in vitro anti-tumour promoting activities of edible plants from Thailand. *Cancer Lett* **95**: 139–146

Murakami A, Nakamura Y, Koshimizu K (1995*b*). Glyceroglycolipids from *Citrus hystrix*, a traditional herb in Thailand, potently inhibit the tumour promoting activity of 12-O-tetradecanoylphorbol-13-acetate in mouse skin. *J Agric Food Chem* **43**: 2779–2783

Murakami A, Ohigashi H and Kohimizu K (1996) Anti-tumour promotion with food phytochemicals: a strategy for cancer chemoprevention. *Biosci Biotech Biochem* **60**: 1–8

Nishino H, Konoshima T, Kozuka M, Tokuda H, Nishino A, Takayasu J, Hasegawa T and Iwashima A (1990) Anti-tumour promoter activity of principles in Zingiberaceae. *J Kyoto Pref Univ Med* **99**: 506

Ohigashi H, Takamura H, Koshimizu K, Tokuda H and Ito Y (1986) Search for possible anti-tumour promoters by inhibition of 12-O-tetradecanoylphorbol-13-acetate induced Epstein–Barr virus activation; ursolic acid and oleanolic acid from an anti-inflammatory Chinese medicinal plant, *Glechoma hederaceae* L. *Cancer Lett* **30**: 143–151

Ohigashi H, Sakai Y, Yamaguchi K, Umezaki I and Koshimizu K (1992) Possible anti-tumour promoting properties of marine algae and in vivo activity of *Wakame* seaweed extract. *Biosci Biotech Biochem* **56**: 994–995

Pitot HC and Dragan YP (1991) Facts and theories concerning the mechanisms of carcinogenesis. *FASEB J* **5**: 2280–2286

Pitot HC and Sirica AE (1980) The stages of initiation and promotion in hepatocarcinogenesis. *Biochim Biophys Acta* **605**: 191–215

Rogers AE, Ziesel SH and Groopman J (1993) Diet and carcinogenesis. *Carcinogenesis* **14**: 2205–2217

Tokuda H, Ohigashi H, Koshimizu K and Ito Y (1986) Inhibitory effects of ursolic and oleanolic acid on skin tumour promotion by 12-O-tetradecanoylphorbol-13-acetate. *Cancer Lett* **33**: 279–285

Towbin H, Staehelin T and Gordon J (1979) Electrophoretic transfer of proteins from polyacrylamide gels to nitrocellulose sheets: procedure and some applications. *Proc Natl Acad Sci USA* **76**: 4350

Wattenberg LW (1985) Chemoprevention of cancer. *Cancer Res* **45**: 1–8

© Cancer Research Campaign 1999

Watternberg LW (1992) Inhibition of carcinogenesis by minor dietary constituents. *Cancer Res* **52**: 2085–2091
Weinstein IB (1991) Cancer prevention: recent progress and future opportunities. *Cancer Res* **51**: 5080–5085
Yadav M, Ilham M and Norhanom W (1989) Epstein–Barr virus early antigen induction in Raji cells by plants used in Malaysian Traditional Medicine. *Asian J Clin Sci* **9**: 71–77
Ziegler RG, Subar AF, Craft NE, Ursin G, Patterson BH and Graubard BI (1992) Does β-carotene explain why reduced cancer risk is associated with vegetables and fruits intake? *Cancer Res* **52**: 2060–2066
Zur Hausen H, O'Neil FJ and Freese UK (1978) Persisting oncogenic herpesvirus induced by the tumour promoter TPA. *Nature* **272**: 373–375
Zur Hausen H, Bornkamm GW, Schmidt R and Hecker H (1979) Tumour initiators and promoters in induction of Epstein–Barr virus. *Proc Natl Acad Sci* USA **76**: 782–785

 *© Cancer Research Campaign 1999*
